# Narrow-Line Width Emission and Room Temperature Amplified Spontaneous Emission in Three-Dimensional Ge Halide Perovskites

**DOI:** 10.1021/acs.chemmater.6c00408

**Published:** 2026-07-03

**Authors:** Marta Morana, Andera Olivati, Marco Moroni, Giulia Folpini, Mehmet Baskurt, Margherita Garbaccio, Annamaria Petrozza, Francesco Ambrosio, Julia Wiktor, Lorenzo Malavasi

**Affiliations:** † Department of Earth Sciences, 9300 University of Firenze, 5012 Firenze, Italy; ‡ Center for Nano Science and Technology, Istituto Italiano di Tecnologia, 20134 Milano, Italy; § Department of Chemistry and INSTM, 19001 University of Pavia, 27100 Pavia, Italy; ∥ Institute for Photonics and Nanotechnology, 96976 CNR − IFN, 20133 Milano, Italy; ⊥ Department of Physics and Astronomy, 11248 Chalmers University of Technology, 41296 Gothenburg, Sweden; # Dipartimento di Scienze di Base e Applicate (DiSBA), 19006 Università degli Studi della Basilicata, 85100 Potenza, Italy

## Abstract

We report the structural and optoelectronic properties of lead-free CsGeI_3_ and CsGeBr_3_ perovskites, unveiling the critical role of local symmetry distortions in defining their emission properties. CsGeBr_3_ exhibits broad photoluminescence from self-trapped excitons, due to local octahedral distortion and a large distribution of the average bond lengths. On the contrary, by using temperature-dependent pair distribution function analysis and hybrid-functional molecular dynamics simulations, we demonstrate that CsGeI_3_ adopts a monoclinic local structure responsible for its narrow near-infrared (NIR) emission (∼745 nm, FWHM ≈ 110 meV at room temperature), the narrowest reported for Ge-based perovskites and in line with tin iodide perovskites. Notably, the high level of structural order also supports the achievement of amplified spontaneous emission (ASE) at room temperature with an exceptionally low threshold (75 μJ/cm^2^), positioning it as a promising candidate for lead-free NIR light-emitting and laser applications.

## Graphical abstract

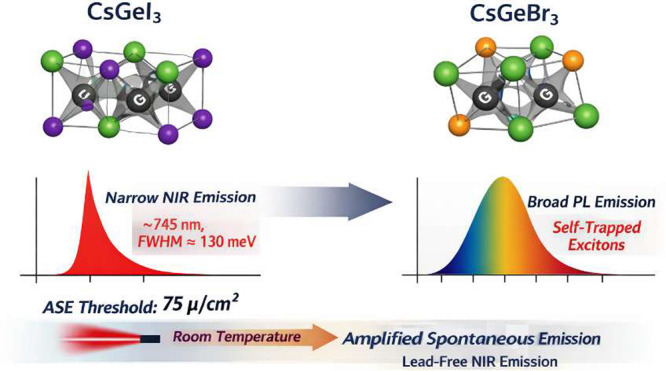

## Introduction

Since they were first proposed as sensitizers for photovoltaic cells,^1^ metal halide perovskites (MHPs) have attracted continuous interest thanks to their promising physical properties.^2–4^ These compounds are described by the chemical formula ABX_3_, where A is Cs or a small organic cation, B is a divalent metal cation, mostly lead, and X is a halide. The chemical composition strongly influences the crystalline and electronic band structures of MHPs, leading to a great variety in optoelectronic properties. Whereas many studies have focused on the role of different A-site cations and halide anions, the B-site cation, which most significantly affects the electronic structure of the semiconductor, has received less attention. Over the past decade, as an alternative to lead, tin halide perovskites have been the subject of intensive research, and more recently, attention has also turned to germanium-based perovskites.^5–12^ The substitution of lead with tin leads to a narrowing of the semiconductor band gap, entering the near-infrared (NIR) spectral region by extending emission wavelengths to ∼1000 nm, effectively covering the biological transparency window (650–950 nm) and the telecom window (800–900 nm). Near-infrared light-emitting sources have garnered significant interest for diverse applications, including night vision, biomedical treatments, optical communication, and data storage.^13,14^ Currently, commercial sources utilize epitaxial heterostructures of III–V inorganic semiconductors as emitters, but, despite achieving nearly 100% internal quantum efficiency, they face limitations stemming from complex material processing and device architectures and, overall, the difficulty of integration in multicomponent photonic and electronic systems. Tin-halide perovskites have emerged as outstanding light-emitting materials due to their simple and versatile synthesis and exceptional optoelectronic properties. However, this class of perovskite still suffers from structural instability and poor control of the p-type self-doping, which leads to broad emission line width and nonradiative recombination of photo-carriers. Germanium halide perovskites share the same advantages of their tin counterparts, in particular, their great optoelectronic properties and the possibility of emission in the NIR region. In fact, germanium iodide-based perovskites show a photoluminescence peak in the spectral range from 650 to 800 nm.^14,15^ Despite the good spectral range of operation of this class of materials, the Ge^2+^ cation has a smaller radius compared to lead and, for this reason, Ge-based perovskites often crystallize, at room temperature (RT), in a lower symmetry space group, usually *R*3*m* (No. 160), with respect to the *Pm*-3*m* (No. 221) of the perovskite aristotype.^16–18^ Such an effect has been correlated to the expression of the ns^2^ lone pair, more significant with a lower atomic number,^19–23^ which becomes particularly evident in Ge-containing compounds where the off-centering of the B cation along the [111] direction induces significant distortions, leading to broad emission.^5,7^ Such a close correlation between lone pair activity, structural distortion, and optoelectronic properties has been investigated, both at the average and local scales, for tin-based perovskites. For example, the local distortions generated by the activity of the lone pair in FASnI_3_ [FA = formamidinium [HC­(NH_2_)^2+^] produce its typical broad energy distribution of the photoluminescence maximum.^24^ On the other hand, despite the increasing interest in lead-free MHPs, detailed correlations of the average and local structure with the optoelectronic response are still lacking for the Ge-containing perovskites even if it is expected that the ease of distortion, due to the presence of the Ge^2+^ cation, may lead to a great tunability of the optical properties. For example, in the case of germanium iodide perovskites, unlike the lead and tin counterparts, changing the cation from Cs to methylammonium (MA) and to the bulkier acetamidinium leads to a large shift in the band gap of almost 1 eV (from 1.6 to 2.5 eV), while maintaining a rhombohedral perovskite structure.^17^ A similar behavior is observed when changing the halogen atom from the larger iodide (I^–^) to bromide (Br^–^) and chloride (Cl^–^), keeping the same cesium (Cs^+^) cation. This halide compositional tuning results in band gap of 1.6, 2.3, and 3.4 eV, respectively,^25^ which is also mirrored in an abundance of different emission peak positions and shapes.

To deepen the comprehension of the role of structural distortion on the optical properties of Ge-based perovskites, here we investigated the average and local structures and optical properties of CsGeBr_3_ and CsGeI_3_ as a function of temperature. The former shows a broad emission centered at 640 nm attributed to self-trapped exciton recombination, while the latter has a very narrow emission centered at 740 nm, making it a great candidate as an NIR emitter material. Moreover, CsGeI_3_ perovskite crystallites show RT amplified spontaneous emission with a very low threshold (75 μJ/cm^2^), comparable to that of the much more studied LHP.

## Results and Discussion

### Local Structure

CsGeI_3_ and CsGeBr_3_ polycrystalline samples have been synthesized according to the procedure reported in the Experimental Section in the Supporting Information (SI). RT laboratory X-ray diffraction confirms their single-phase nature (see Figure S1). In reciprocal space, temperature-dependent synchrotron X-ray diffraction data show that both CsGeI_3_ and CsGeBr_3_ crystallize in space group *R*3*m* in the whole temperature range investigated, in good agreement with previous literature reports (Figure S2). Moving into direct space and to the local scale, a visual inspection of the Pair Distribution Functions (PDFs) up to 5 Å from RT down to 90 K shows two peaks in the range of the Ge-X distances, consistent with a symmetry lower than cubic, such as the rhombohedral one (Figure 1).

**Figure 1:**
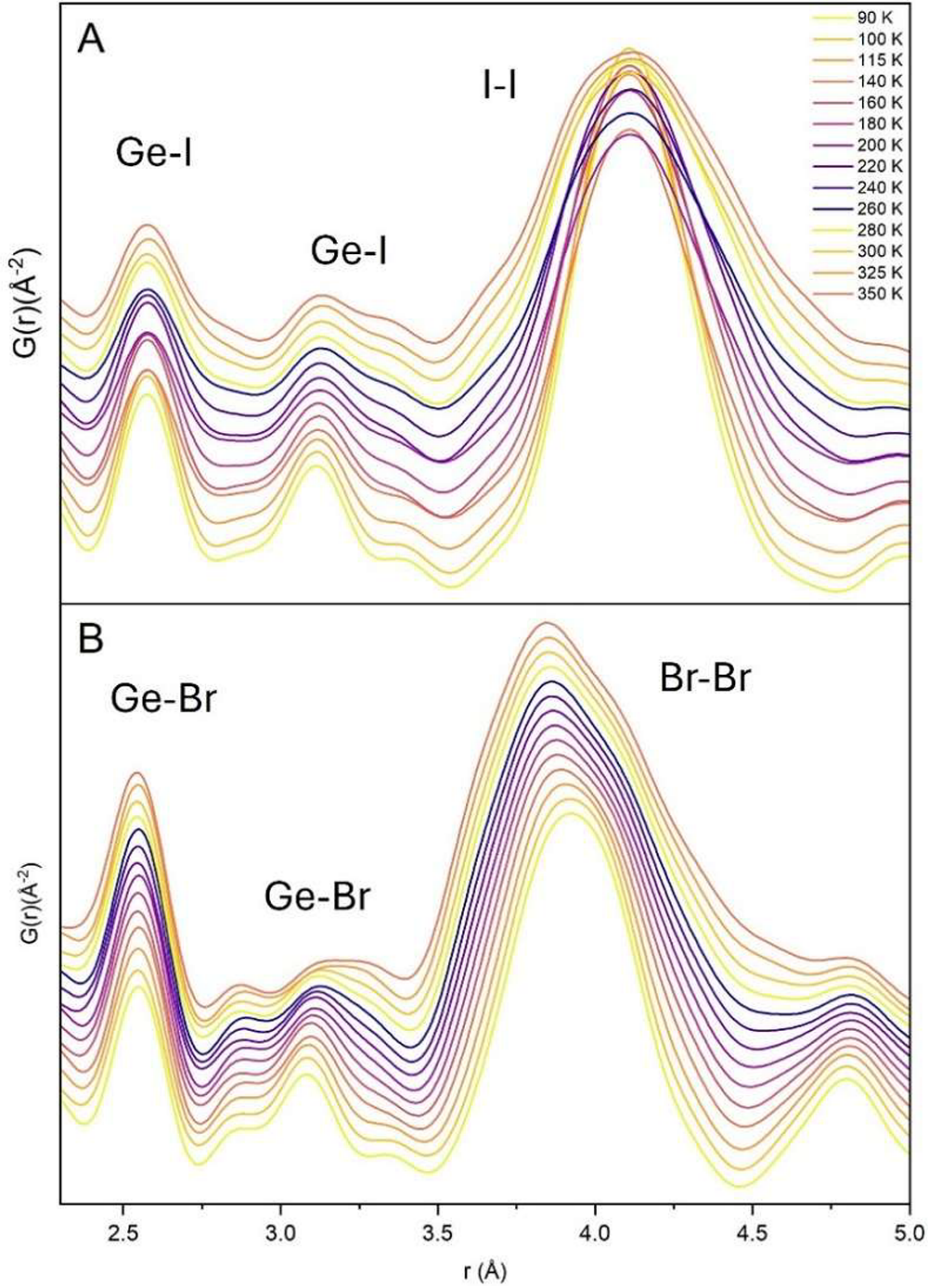
Overlay of the raw X-ray PDF data collected from 90 to 350 K for (A) CsGeI_3_ and (B) CsGeBr_3_.

Fitting of the RT PDF data of the iodide and bromide compounds in the *R*3*m* space group shows a relatively good agreement (see Figure 2a,c). It is worth noting that this space group was also proposed to describe the local structure of FAPbBr_3,_ ^22^ MASnI_3_,^22,26^ FASnI_3_,^22,26^ and several all-inorganic titanates.^27–29^ However, this symmetry does not allow for a good description of the shoulder visible on the second peak related to the Ge–X distances, more markedly in the iodide compound (Figure 2a, see arrow), suggesting that the symmetry at the local scale might be lower. Group-subgroup relationships identify space group *Cm* as a possible alternative. In space group *R*3*m,* the GeX_6_ octahedra contain two groups of distances, whereas the lower monoclinic symmetry allows for four potential distributions of Ge–X bond lengths. A sketch of the rhombohedral and monoclinic unit cells is reported in Figure 2e,f, respectively.

**Figure 2:**
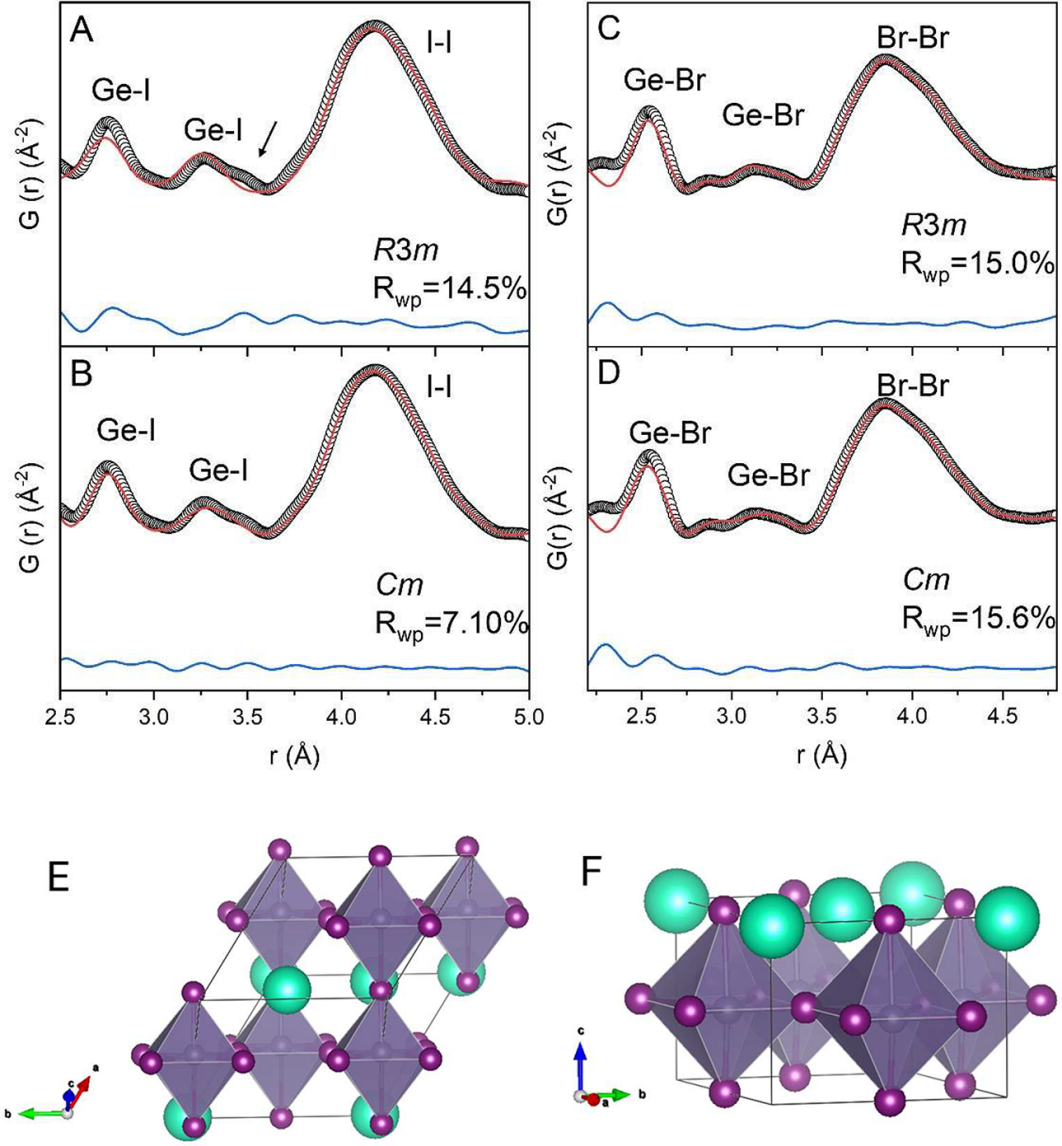
Fits of the RT X-ray PDF data for CsGeX_3_ against space groups *R*3*m* and *Cm* for X = I (A, B) and X = Br (C, D). The arrow marks the shoulder on the second Ge–I distance described in the text. Sketches of the rhombohedral (E) and monoclinic (F) unit cells.

As a matter of fact, the fitting of RT PDF data for CsGeI_3_ against the monoclinic space group provides good agreement (see Figure 2b). Such symmetry properly describes the PDF data of CsGeI_3_ also at low temperatures, as reported in Figure 3a.

**Figure 3:**
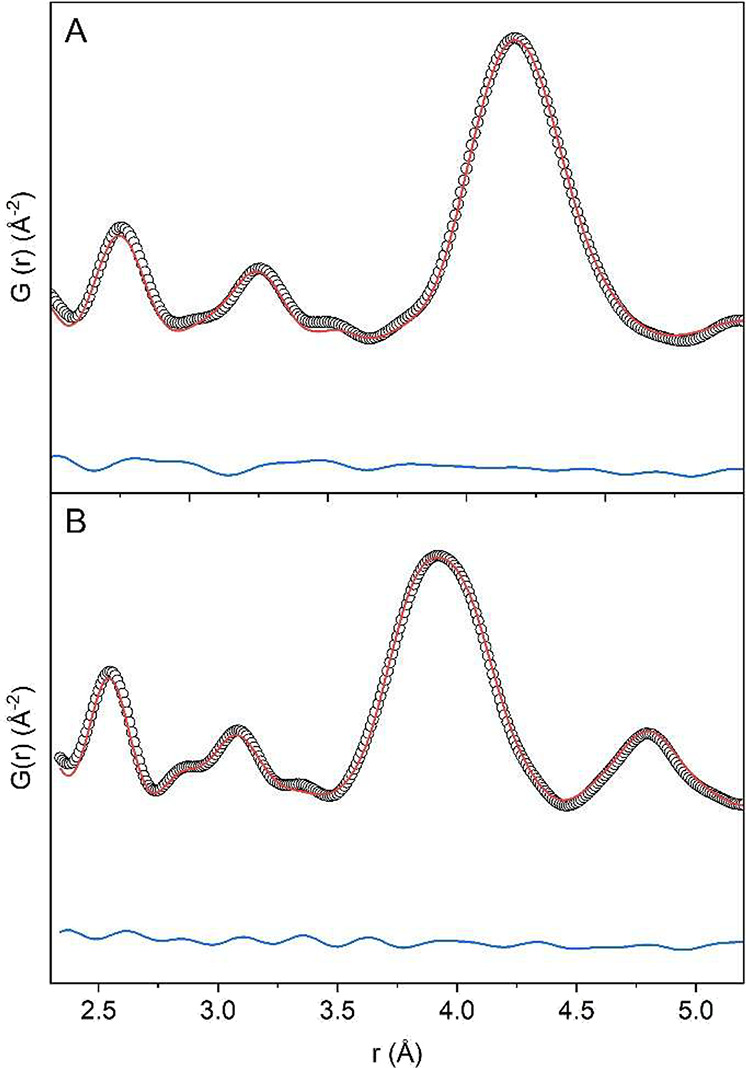
Fits of the X-ray PDF data for (A) CsGeI_3_ against space groups *Cm* at 90 K (*R*_wp_ = 6.25%) and for (B) CsGeBr_3_ against space groups *R*3*m* at 90 K (*R*_wp_ = 6.72%).

On the other hand, at RT, CsGeBr_3_ shows a less marked preference for the monoclinic symmetry, and fits against the *R*3*m* and *Cm* space groups provide similar agreement, with *R*_wp_ values of 15.0 and 15.6%, respectively (Figure 2c,d). *R*3*m* symmetry well describes the PDF of CsGeBr_3_ even at 90 K (Figure 3b). It is important to note, however, that the bond-length distribution of the Ge–X octahedra appears to be broader for the CsGeBr_3_ with respect to CsGeI_3,_ even though it is limited to two groups of bond lengths. This can be qualitatively assessed by looking at the second group of peaks in Figure 1. Notably, ab initio calculations showed that the root-mean-square-displacement (RMSD) of Ge in these compounds is greater for I than Br,^30^ which could explain the slightly smaller distortion in the bromide MHPs. It appears that CsGeX_3_ MHPs have a different general trend with respect to Pb and Sn compounds. In fact, these compounds usually adopt, at the local scale, a lower symmetry with respect to that of the average structures, whereas this tendency appears to be less significant for the Ge-containing counterparts. At lower temperatures, the data from CsGeI_3_ cannot be fitted against the rhombohedral space group, but only against the monoclinic one (*Cm*) as shown in Figure 3a. This behavior is in good agreement with other MHPs that showed a trend of increasing distortion at the local scale upon cooling. It is worth noting that FAGeBr_3_ was reported to crystallize in *Cm* at 100 K and that the local structure of several hybrid perovskites was reported to have the same symmetry as their low temperature average structure, namely MAPbBr_3_,^31,32^ MAPbCl_3_,^33^ MASnBr_3_,^26^ and FASnBr_3_.^26^ To complement the experimental total scattering results, we performed hybrid-functional molecular dynamics (MD) simulations at 100, 200, and 300 K for CsGeI_3_ and CsGeBr_3_. The simulated PDFs reproduce the experimental trend of temperature-induced broadening in the Ge–X and X–X correlations (Figure 4). A further comparison between the experimental and MD-calculated PDFs is reported in Figure S3. To this end, we selected 20 snapshots separated by 200 fs from each trajectory and calculated the average *G*(*r*) plots for each material. The comparison between these average calculated PDFs and the experimental data at RT for the two compounds is given in Figure S3, where only the scale factor and the unit cell parameters have been refined. Overall, the calculated and experimental structures fairly agree in terms of peak number, shape, and positions, supporting the validity of the computational modeling and strongly indicating that a proper description of the energy gaps in Ge halide perovskites requires the consideration of the local structural distortions experimentally unveiled by pair distribution function analysis. Such an approach allowed us to highlight the local symmetry characteristics of the two Ge perovskites, with the iodide sample described in the full temperature range with a monoclinic structure and the bromide sample showing a rhombohedral symmetry at 90 K and a monoclinic or rhombohedral symmetry at RT. Importantly, it appears that, the bond-length distribution of the CsGeBr_3_ sample is broader than that of the iodide counterpart.

**Figure 4:**
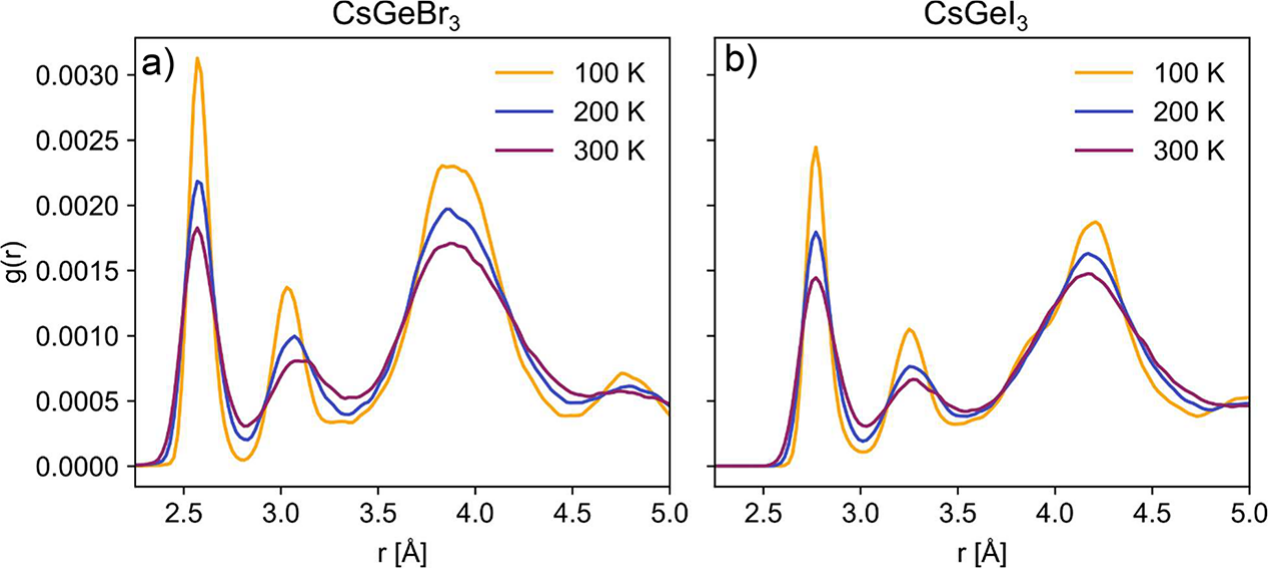
Calculated radial distribution functions from MD simulations for CsGeX_3_, X = I, Br at 100, 200, and 300 K.

Although all structural and optical measurements were conducted under an inert atmosphere, the stability of the samples was systematically investigated in light of the well-known tendency of germanium-based perovskites to undergo oxidation and degradation under ambient conditions, driven by the oxidation of Ge^2+^.^7^ Laboratory powder XRD patterns have been collected on CsGeI_3_ and CsGeBr_3_ samples left open to the laboratory environment (about 20 °C and 30% relative humidity) as a function of time up to 336 h (Figure S4). Diffraction patterns are reported in Figure S4. It is interesting to note that CsGeBr_3_ retains all of the peaks of the main phase up to the maximum time interval investigated, with the appearance of a few additional peaks (not directly corresponding to known oxidized phases). On the other hand, CsGeI_3_ is significantly less stable, with a relevant decomposition of the perovskite phase occurring already after 48 h.

### Optical Properties

Following the elucidation of the average and local structures of CsGeI_3_ and CsGeBr_3_ as a function of temperature, coupled with MD simulations, the optical properties of the two samples in powder form have been investigated by means of photoluminescence (PL) and absorption spectroscopies.

At RT, CsGeBr_3_ has a band gap of 2.36 eV (525 nm) (see Figure S5) and shows a featureless and broad emission spectrum centered at 640 nm spanning through the entire visible range, with a full width at half-maximum (FWHM) of about 150 nm and a considerable Stokes shift (∼100 nm). Such a kind of emission is usually ascribed to defect-mediated or self-trapped exciton recombination (STE). To further elucidate the origin of the optical properties of CsGeBr_3_, we carried out low-*T* PL measurements as reported in Figure 5a. Upon decreasing the temperature down to 205 K, the emission intensity increases and the fwhm decreases, as expected. When reducing the density of phonons, some nonradiative pathways are hindered and the broadening of the emission peak is reduced. Further reducing the temperature down to 77 K, the peak position continues to linearly redshift but changes its slope, on the contrary the FWHM increases (Figure S6). This observation is in accordance with the PDF results reported above, highlighting a potential difference between the distortion degree at RT and at cryogenic temperatures. Additional evidence for this structural change is provided by the sample color change from ocher at RT to dark red at 77 K, as shown in the inset in Figure S6. Moreover, at 175 K, a narrow peak (FWHM ∼ 13 nm) related to excitonic recombination emerges and becomes the dominant emission at 77 K.

**Figure 5:**
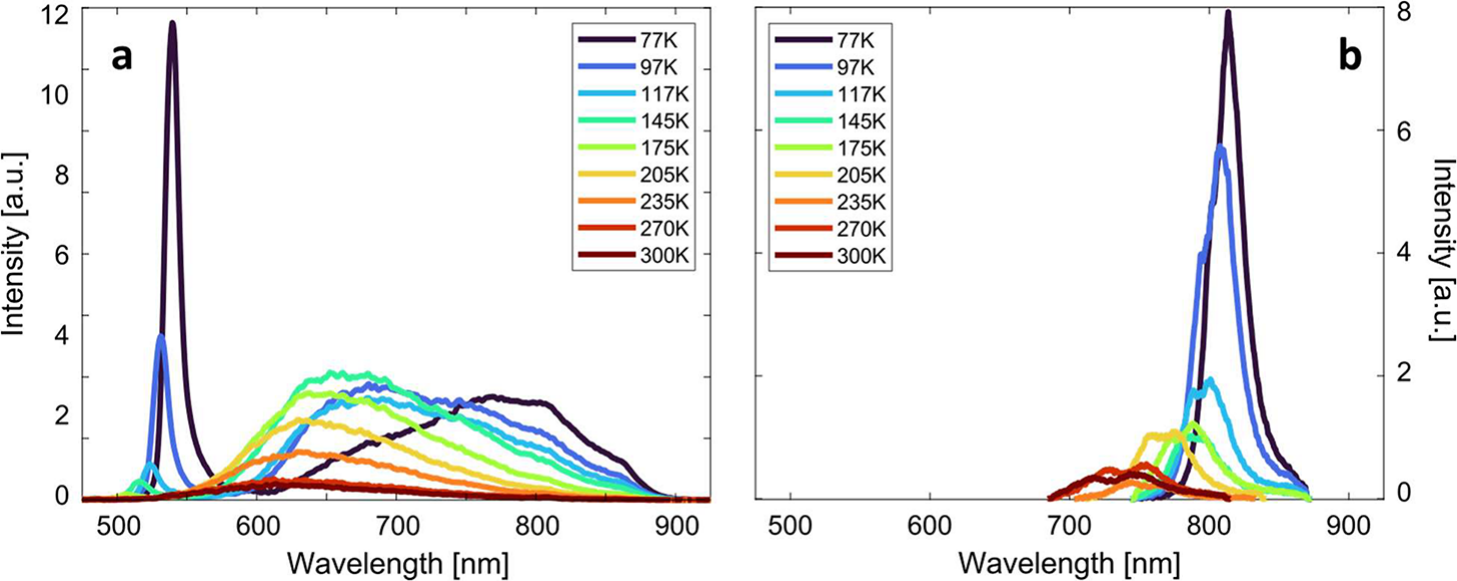
Photoluminescence spectra of (a) CsGeBr_3_ and (b) CsGeI_3_ powder samples as a function of temperature from RT to 77 K.

To identify the nature of the broad emission in CsGeBr_3_, we performed excited-state calculations which found a stable self-trapped exciton (STE) with a formation energy of −0.41 eV in the material (see Figure 6), consistent with the STE stability previously reported for CsGeBr_3_ using hybrid density functional theory.^34^ The STE corresponds to the localization of the electron and hole on neighboring Ge-centered octahedra. This localization induces a strong local lattice relaxation, with Ge–Br bonds elongating around the electron-trapping site and contracting around the hole-trapping site. Such a displaced excited-state minimum naturally leads to a large Stokes shift and broad emission, as radiative recombination occurs from a strongly relaxed geometry and is therefore accompanied by significant vibronic broadening. Using the relaxed STE geometry, we estimate the emission energy from the total-energy difference between the triplet and singlet states evaluated at the STE geometry (see SI for details).

**Figure 6:**
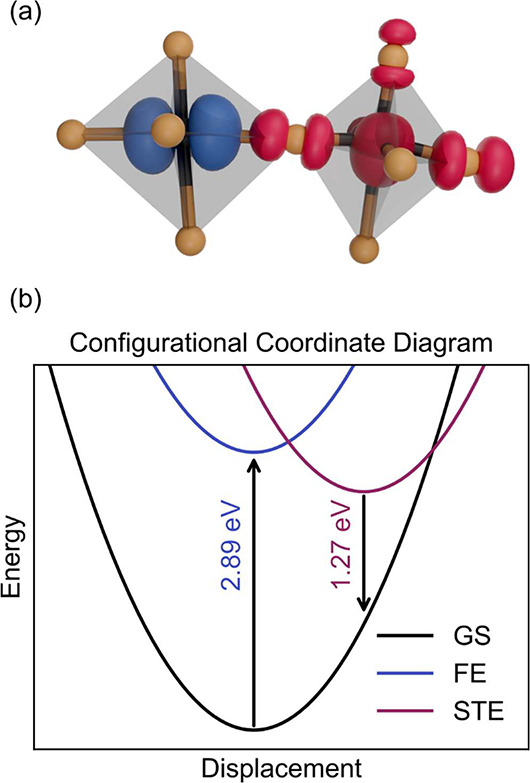
(a) Self-trapped exciton (STE) configuration in CsGeBr_3_ from hybrid-DFT excited-state calculations, showing electron (blue) and hole (red) localization on neighboring Ge-centered octahedra and the associated local lattice relaxation. (b) Configurational-coordinate diagram illustrating vertical absorption and emission energies; the emission energy is estimated from the singlet–triplet total-energy difference evaluated at the relaxed STE geometry.

Changing the anion from bromine to iodine greatly alters the emission properties (see Figure 5b). At RT, the emission, centered at 745 nm, is characterized by a narrow peak (FWHM ∼ 70 nm/130 meV) with a minimal Stokes shift (band gap = 1.61 eV/740 nm, Figure S5). The double peak that characterizes the shape of the emission is caused by the self-absorption and multiple reflections of emitted photons in the powder samples.^35^ This is further corroborated by the identical lifetime of the two peaks extracted by time-resolved photoluminescence measurements (TRPL) as observed in Figure S7. Lowering the temperature, the FWHM monotonically decreases to less than 30 nm (50 meV) at 77 K (Figure S8), consistent with the above-reported total scattering data, which indicate that the local monoclinic structure actually describes the octahedral distortion in the whole temperature range. We emphasize that the emission width is the lowest for any germanium-based perovskite both at RT (∼140 meV) and 77 K (∼50 meV). These values are comparable with optimized thin films or single crystals of CsPbI_3_ and lower than those of the tin counterpart, making CsGeI_3_ perovskite a great candidate for light-emitting applications.^12,36^

We note that in CsGeI_3_, the formation of STEs, obtained with excited-state calculations, is not favorable, explaining the lack of Stokes shift in this material and the comparatively narrow emission observed experimentally. This indicates that photoexcited carriers in CsGeI_3_ remain mainly in near-band-edge states rather than relaxing into strongly distorted self-trapped configurations. This is favorable for achieving optical gain since STE formation would act as a loss channel by localizing carriers and broadening the emission spectrum. In contrast, CsGeBr_3_ hosts a stabilized STE minimum, and using the relaxed STE geometry, we estimate an emission energy of 1.27 eV at 0 K (see Figure 6). This value is lower than the emission energy extrapolated from the experimental spectra to 0 K (≈1.55 eV), which can be rationalized by the approximate nature of the computational estimate: the calculated value corresponds to a vertical singlet–triplet energy difference evaluated at the triplet geometry and therefore neglects nuclear quantum effects (zero-point motion) and finite-temperature dynamic disorder contributions that are expected to renormalize the excited-state potential energy surfaces. In addition, small systematic errors associated with the electronic-structure description of localized excited states (e.g., exchange–correlation functional dependence) can contribute to residual offsets. Despite this quantitative difference, the calculations capture the key qualitative trend, namely that STE formation is energetically favored in CsGeBr_3_ but suppressed in CsGeI_3_, providing a rationale for the broad, strongly Stokes-shifted emission of the bromide and the narrow, near-band-edge emission of the iodide observed in experiments.

The temperature evolution of the band gap was also extracted from the MD simulations and follows the experimental trend (Figure S9), with CsGeBr_3_ showing a stronger blueshift with an increase in temperature. This trend can be seen also by monitoring the emission position of the two samples in the same temperature range analyzed: in the case of CsGeBr_3_ perovskite, we can see a shift of the broad peak of about 250 meV, while for the CsGeI_3_ sample the shift is reduced to 150 meV (see Figure 5). The lower shift of the band gap upon lowering the temperature, in addition to the very narrow emission, suggests a low amount of defects and a low coupling with phonons.

To further investigate the optical properties of CsGeBr_3_ and CsGeI_3_, we performed fluence-dependent measurements at different temperatures in a fluence range from 10^16^ to 10^20^ absorbed photons/cm^3^. To address the origin of the emission of the bromine sample, we fit the fluence dependence of the two emission peaks with a power law, *I* = *P^K^*, as shown in Figure S10. Fitting the measurement taken at 97 K as a reference, we obtained values of 1.005 and 0.7915 for the narrow and broad emissions, respectively. A value of the exponential *K* close to 1 suggests excitonic recombination, while a lower value is related to defect-mediated recombination or self-trapped excitons. This, in addition to the lattice distortion due to Ge substitution and the above-reported excited-state calculations, corroborates the idea of excitonic and defect-mediated/STE recombination for the narrow and broad emissions from CsGeBr_3_, respectively.^37,38^

The CsGeI_3_ sample, in the temperature range from 300 to 235 K, shows a superlinear behavior vs fluence, suggesting a gain process in the material (Figure S11). As shown in Figure 7a, by further lowering the temperature, a narrow peak (FWHM 4–8 nm) starts to appear at 77 K, centered at 815 nm. As can be seen from Figure 7b, its intensity rises exponentially with the incident fluence, suggesting an amplified spontaneous emission (ASE) process.

**Figure 7:**
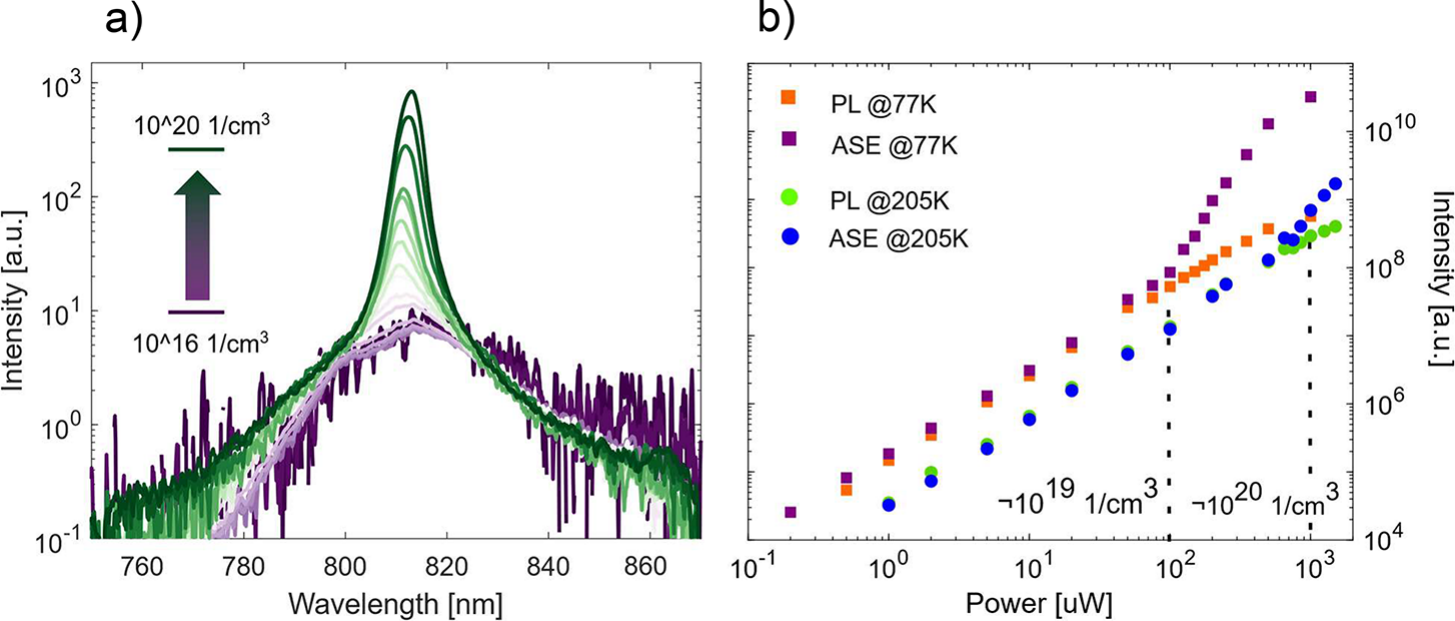
(a) PL spectra of CsGeI_3_ sample taken at 77 K for different laser fluences from 10^16^ to 10^20^ photons/cm^3^ and (b) power dependent emission intensity for the narrow (ASE) and broad (PL) peak at different temperatures: 77 K (violet and orange and green) and 205 K (blue and green).

Figure 7b shows the comparison of the intensity of the broad emission and the narrow ASE peak vs incident light power at 205 and 77 K. The point from which the ASE peak starts to have an exponential trend is the threshold for ASE. As we can notice, the threshold decreases from 10^20^ (4300 μJ/cm^2^) to 10^19^ (430 μJ/cm^2^) upon lowering the temperature down to 77 K. We stress the fact that these results were obtained by measuring powder samples, in which the problem of reabsorption and a huge amount of scattering usually make it really difficult to achieve ASE.^39^

Driven by these encouraging results, we moved to deposit CsGeI_3_ on a glass substrate through a one-step deposition (see SI). The crystal structure is confirmed by XRD measurements (Figure S12), with the main peaks corresponding to the calculated average rhombohedral structure *R*3*m* as reported above. Scanning electron microscopy (SEM) images (Figure S13) show that CsGeI_3_ does not cover the substrate uniformly, but the sample is composed of large crystalline islands of perovskite measuring tens of μm, together with smaller grains. Having proven the right composition and structure, we performed absorption and fluence dependence PL measurements at RT over a fluence range of more than 2 orders of magnitude.

The absorption edge of the CsGeI_3_ crystallites deposited on glass is comparable to that of the powder sample, 1.64 eV, as shown in Figure S14. Moreover, looking at the emission spectra shape (Figure S15), we can notice that the emission of the perovskite crystallites overlaps with that of the powder sample and that the second peak intensity is greatly reduced, as expected, due to the decreased reabsorption and scattering, revealing a FWHM of 110 meV (∼50 nm). As we can observe from Figure 8a, by increasing the fluence on the sample, a narrow ASE peak appears at 748 nm. From the trend of the FWHM and peak intensity at 748 nm by increasing the incident fluence, it is possible to extrapolate the threshold of this phenomenon. At the threshold of ASE, the FWHM of the dominant emission decreases from more than 30 nm to 4–6 nm and the intensity at 748 nm (red dot in Figure 8b) starts rising exponentially, while the broad peak (blue dots) follows a linear trend. The value at which this occurs is around 75 μJ/cm^2^, which is an incredibly low value for a novel material, even compared to the lead and tin counterparts, whose threshold usually lie in a range between 0.5 and hundreds of μJ/cm^2^ for optimized deposition, passivation, and measurement conditions.^40^ To the best of our knowledge, this is the first reported measured ASE in a fully inorganic germanium perovskite. Further investigations on the deposition method are needed in order to obtain a uniform layer of perovskite, thereby reducing roughness and defectivity.

**Figure 8:**
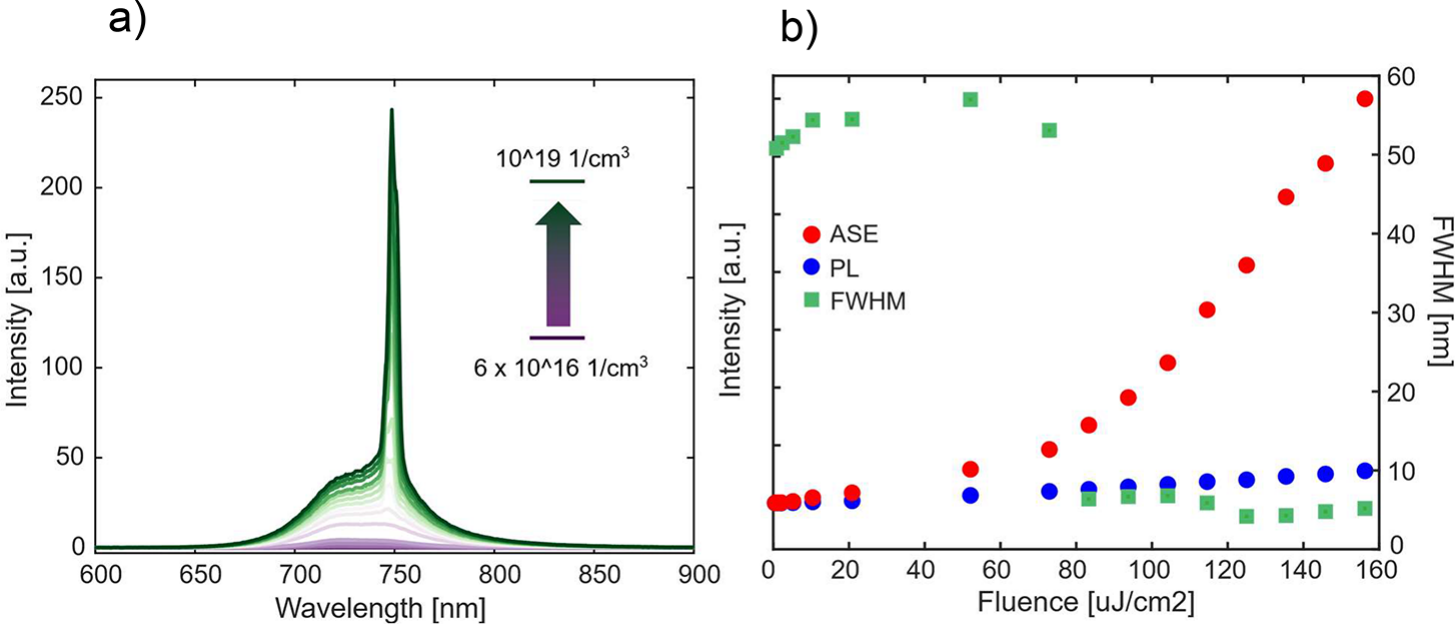
(a) Fluence dependent PL spectra of CsGeI_3_ crystallites at room temperature. (b) Fluence dependent emission intensity for the narrow (ASE, red dot), broad (PL blue dot), and fwhm (green square) of the dominant emission.

Taken together, our structural, computational, and optical results show that emission in CsGeX_3_ is governed not by average crystallographic symmetry alone, but by the topology of the local distortion landscape created by the Ge^2+^ off-centering. In CsGeI_3_, PDF analysis and hybrid-functional MD reveal a coherent symmetry lowering to a monoclinic local motif that persists over the investigated temperature range and is associated with a comparatively narrow Ge–I bond-length distribution. Thus, the lone-pair-driven distortion is accommodated within a single local geometry, so that photoexcitation requires only limited further lattice reorganization. Consistent with the minimal Stokes shift, the smaller temperature-induced spectral renormalization, and the absence of a favorable self-trapped exciton minimum in the excited-state calculations, the emissive state remains predominantly near-band-edge and only weakly coupled to the lattice, thereby enabling narrow line widths and optical gain. By contrast, CsGeBr_3_ exhibits a broader Ge–Br bond-length distribution despite its nominally higher local symmetry. In this case, the excited state couples more strongly to lattice distortions and relaxes into a localized self-trapped exciton, yielding the large Stokes shift and STE-like fluence dependence observed experimentally. The key design rule that emerges is that narrow-band lead-free perovskite emitters should be sought among compositions that enforce a single, well-defined local distortion with low bond-length variance and no stabilized self-trapped excited-state minimum, whereas broad emitters will arise when symmetry breaking is spatially distributed and accompanied by strong excited-state lattice relaxation. This predicts that the best narrow-band candidates will combine narrow PDF/MD bond-length histograms, weak temperature-dependent line width or band-edge renormalization, and the absence of persistent carrier localization in excited-state calculations.

## Conclusions

In this work, we have explored the structural and optical properties of the lead-free germanium halide perovskites CsGeI_3_ and CsGeBr_3_. Both compounds crystallize in the rhombohedral space group *R*3*m*, but total scattering and PDF analysis indicate a lower symmetry at the local scale, capable of describing the observed broad distribution in the Ge–X bond lengths. While for CsGeI_3_ a monoclinic structure well describes the local structure in the whole temperature range, in the case of CsGeBr_3_ a possible variation of the local structure has been found from RT to low temperatures, coupled to a broader Ge–X bond distribution. The analysis of the optical response shows that CsGeBr_3_ exhibits broad, featureless photoluminescence centered at 640 nm with a large Stokes shift (∼100 nm) and a FWHM of 150 nm, indicative of defect-mediated or STE recombination. Excited-state calculations confirm the presence of a stable STE with a formation energy of −0.41 eV, and fluence-dependent measurements show a sublinear power-law exponent (*K* ≈ 0.79), further supporting this recombination mechanism. Upon cooling, CsGeBr_3_ we can see the emergence of a sharp excitonic peak (FWHM ∼ 13 nm) and a shrinkage followed by a broadening of the main emission peak in accordance to the preferences of the local symmetry extrapolated from PDF data. In contrast, CsGeI_3_ displays a notably different behavior. At RT, the emission consists of a narrow peak centered at 745 nm with a minimal Stokes shift and a FWHM of ∼110 meV, narrowing to ∼50 meV at 77 K. This emission is the narrowest reported for germanium-based perovskites and rivals that of optimized lead and tin halide perovskites thin films, making this material a promising option for LED applications. Fluence-dependent measurements reveal superlinear behavior in the 300–235 K range, suggesting a gain process and, at 77 K, a narrow ASE peak emerges at 815 nm. The ASE threshold decreases significantly with temperature, from 4300 to 430 μJ/cm^2^. Remarkably, CsGeI_3_ crystallites deposited on glass via a one-step deposition exhibit ASE at 748 nm with a low threshold (75 μJ/cm^2^), which is among the lowest reported for lead-free perovskites. This achievement is particularly notable, making this material a great candidate as a gain material for laser applications.

## Experimental Section

### Synthesis

CsGeI_3_ and CsGeBr_3_ powdered samples were prepared starting from stoichiometric amounts of germanium bromide or iodide and cesium bromide or iodide dissolved in excess HBr or HI, respectively. After heating to 90 °C while stirring, the solution was then heated to 100 °C for 30 min and left to cool at 1 °C/min. CsGeI_3_ deposited on glass/ITO substrates were produced with a one-pot approach. We dissolved 80 μg of CsI and 100 μg of GeI_2_ in 2 mL of DMF, then stirred and heated the solution at 120 °C for 1 h. 20 μL of the solution, after being filtered, was deposited on plasma-treated grass or ITO substrates. As the last step, the samples were placed on a hot plate at 60 °C for 5 min for a fast annealing process. Samples quality was checked by laboratory XRD using a Bruker D6 Cu-anode diffractometer.

### X-ray Total Scattering

X-ray PDF data were collected at the ID11 materials science beamline at ESRF, Grenoble, (λ = 0.158135 Å), using a FReLoN camera. The sample-to-detector distance (105 mm) was calibrated against a CeO_2_ standard using the pyFAI^41^ package, which was also employed to integrate the 2D images. Sample powders were loaded into quartz capillaries (provided by Hilgenberg) with a 0.5 mm diameter, which were spun during data collection. A Cryostream was employed to collect low- and high- temperature data from 80 to 360 K. PDFs were obtained using PDFgetX3 ^42^ with a *Q*_max_ of 26 Å and PDF modeling was carried out using PDFGui.^43^ The instrumental parameters *Q*_broad_ and *Q*_damp_ were set to 0 and 0.046, respectively, as determined from the CeO_2_ standard. Starting coordinates for the various structural models were obtained from literature data and from calculations in ISODISTORT,^44,45^ starting from the reported model and employing “method 1” in the search.

### Optical Measurements

Static and time-resolved photoluminescence measurements were performed by exciting the sample with the third harmonic (355 nm) of a Nd:YAG Picolo-AOT laser (pulse length of 1000 ps, 1 kHz repetition rate). The laser was focused on the sample placed in a cooled Linkam stage under vacuum. The emission of the samples was collected with an Andor iStar 320T ICCD camera coupled to a Shamrock 303i spectrograph.

Absorption measurements on CsGeI_3_ deposited on a glass substrate were performed using a UV–vis spectrometer (PerkinElmer Lambda 1050).

### Crystallites Characterization

XRD measurements on CsGeI_3_ deposited on a glass substrates were performed using a BrukerTM diffractometer equipped with a CuK anode (λ = 1.544060 Å). The samples were kept in a controlled nitrogen environment to prevent degradation. SEM images were acquired with a MIRA3 TESCAN system with an accelerating voltage of 5 kV.

## Computational Details

Ab initio molecular dynamics (MD) simulations were carried out for CsGeBr_3_ and CsGeI_3_ using the CP2K package within the Quickstep module.^46,47^ A hybrid exchange-correlation functional (PBE0-TC-LRC) was used, with the fraction of exact exchange, α determined via the Koopmans’ condition in ref 8: 26% for CsGeBr_3_ and 21% for CsGeI_3_.^48^ The auxiliary density matrix method (ADMM) was employed together with the truncated Coulomb operator (6 Å cutoff), using Goedecker–Teter–Hutter pseudopotentials and MOLOPT basis sets. Simulations were run in the NVT ensemble at 100, 200, and 300 K, using volumes matched to experimental unit cells at each temperature. A Nosé thermostat was applied. Each trajectory consisted of 7–8 ps, with the first 1.5 ps discarded for equilibration. The time step was 5 fs. The average band gap at each temperature was computed from instantaneous HOMO–LUMO gaps sampled every 0.5 ps. Spin–orbit coupling (SOC) corrections to the band gap were calculated using VASP by comparing calculations with and without SOC.^49,50^

To investigate the nature of the broad emission in CsGeBr_3_, we performed excited-state calculations on a monoclinic supercell in the triplet state. The STE was localized by relaxing the triplet state at 0 K with initial distortions around neighboring Ge atoms. The STE localization protocol follows ref 11. The STE formation energy (*E*_f_^STE^) was computed as the adiabatic stabilization of the localized triplet configuration relative to the delocalized excitation:

$$E_{f}^{\mathrm{STE}}=E_{T}^{\mathrm{STE}}-E_{S}^{\mathrm{GS}}-E_{\mathrm{gap}}$$

where *E*_T_^STE^ is the total energy of the relaxed triplet STE configuration, *E*_S_^GS^ is the total energy of the relaxed singlet ground state, and *E*_gap_ is the band gap computed with the same hybrid functional and exact-exchange fraction α (estimated from the Kohn–Sham HOMO–LUMO gap). The emission energy was estimated using a ΔSCF approach as the vertical singlet–triplet energy difference evaluated at the relaxed STE geometry.

## Supplementary Material



## Notes

Experimental and computational methods; additional experimental data (PDF)
