# Supplementary material for: Narrow-Line Width Emission and Room Temperature Amplified Spontaneous Emission in Three-Dimensional Ge Halide Perovskites
Source: Chem Mater. 2026 Jul 3;38(13):6446–55. doi: 10.1021/acs.chemmater.6c00408 (PMC13374735; doi:10.1021/acs.chemmater.6c00408)
Supplement: Supplementary file 1 [file cm6c00408_si_001.pdf]

Narrow-Linewidth Emission and Room Temperature Amplified Spontaneous Emission in Three-Dimensional Ge Halide Perovskites

Marta Morana,[a,◊] *Andera Olivati,*[b,◊] *Marco Moroni,*[c] *Giulia Folpini,*[c,d] *Mehmet Baskurt,*[e] *Margherita Garbaccio,*[c] *Annamaria Petrozza,*[b] *Francesco Ambrosio,*[f] *Julia Wiktor,*[e] *Lorenzo Malavasi,*[c,*]

[a]University of Firenze, Department of Earth Sciences, Firenze, 5012, Italy
[b]Center for Nano Science and Technology@PoliMi, Istituto Italiano di Tecnologia, Milano, 20134, Italy
[c]University of Pavia, Department of Chemistry and INSTM, Pavia, 27100, Italy
[d]Institute for Photonics and Nanotechnology, CNR – IFN, Milano, 20133, Italy
[e]Department of Physics, Chalmers University of Technology, Gothenburg, 41296, Sweden
[f]Dipartimento di Scienze di Base e Applicate (DiSBA), Università degli Studi della Basilicata, Potenza, 85100, Italy

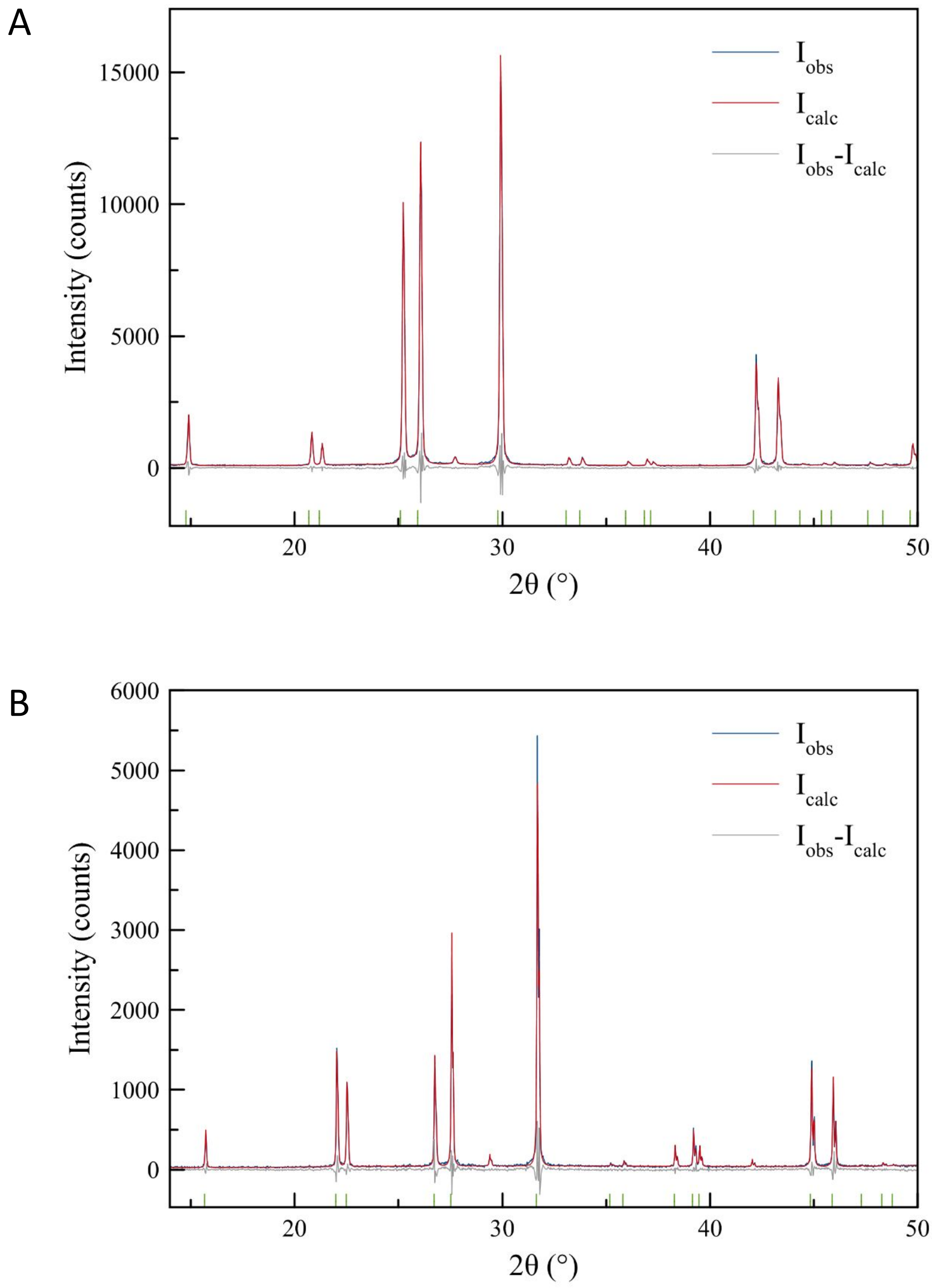

Figure S1. LeBail refined laboratory X-ray diffraction patterns of (A) $CsGeI_3$ and (B) $CsGeBr_3$.

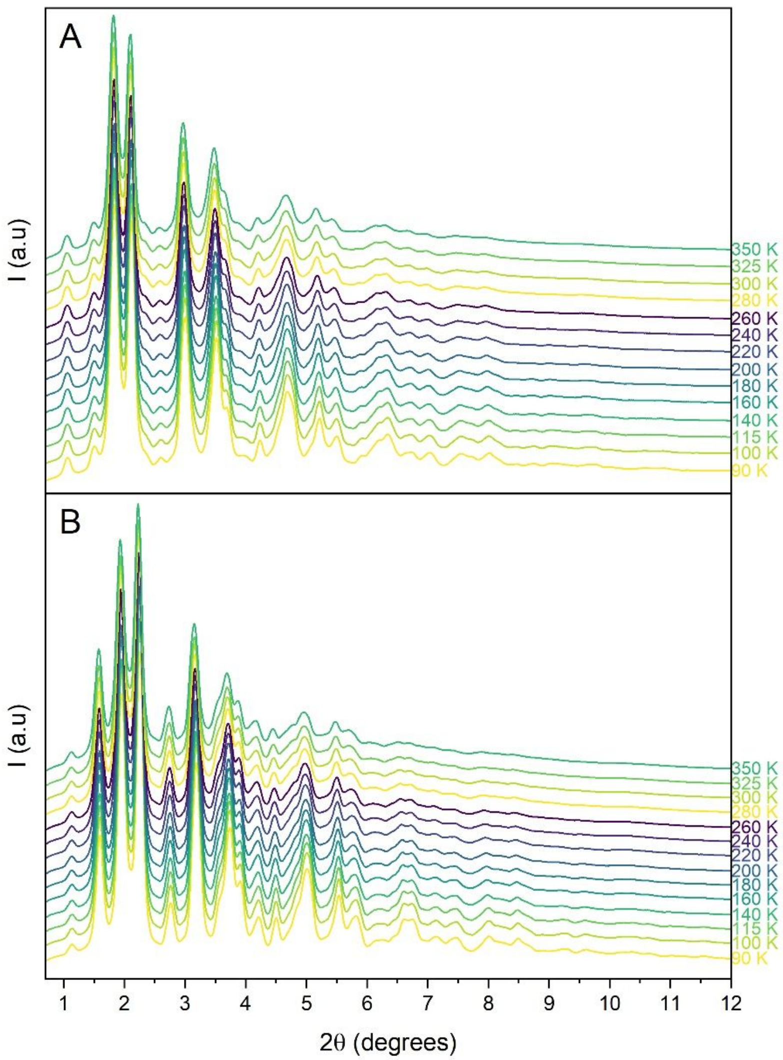

Figure S2. Temperature dependent synchrotron X-ray diffraction patterns of (A) $CsGeI_3$ and (B) $CsGeBr_3$.

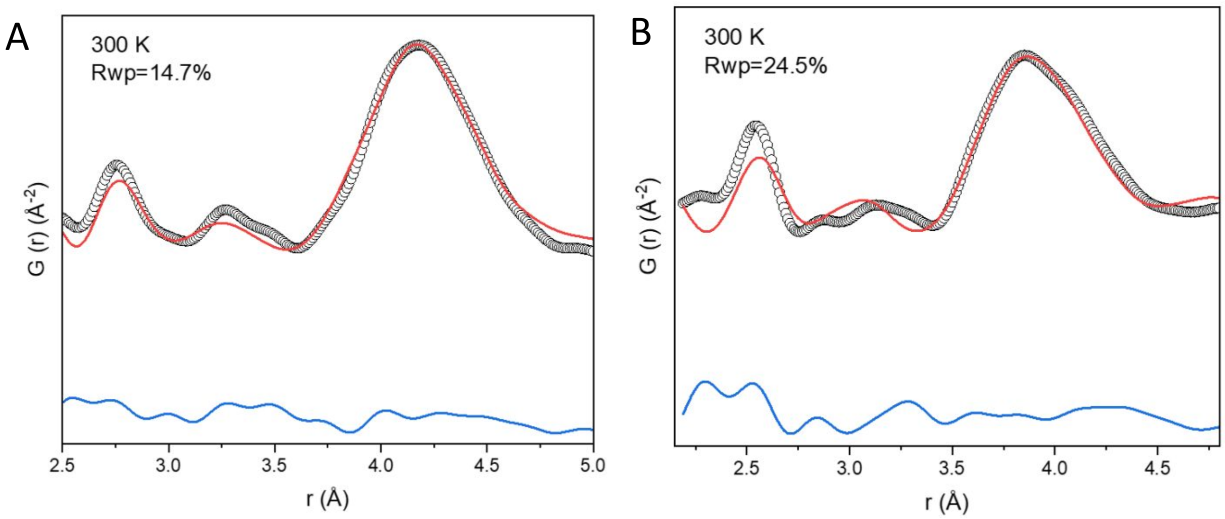

Figure S3. Comparison between X-ray PDF data collected at RT and MD simulations for $CsGeX_3$, X= I (a), Br (b). Gray dotted line: observed; red line: calculated; blue line: difference.

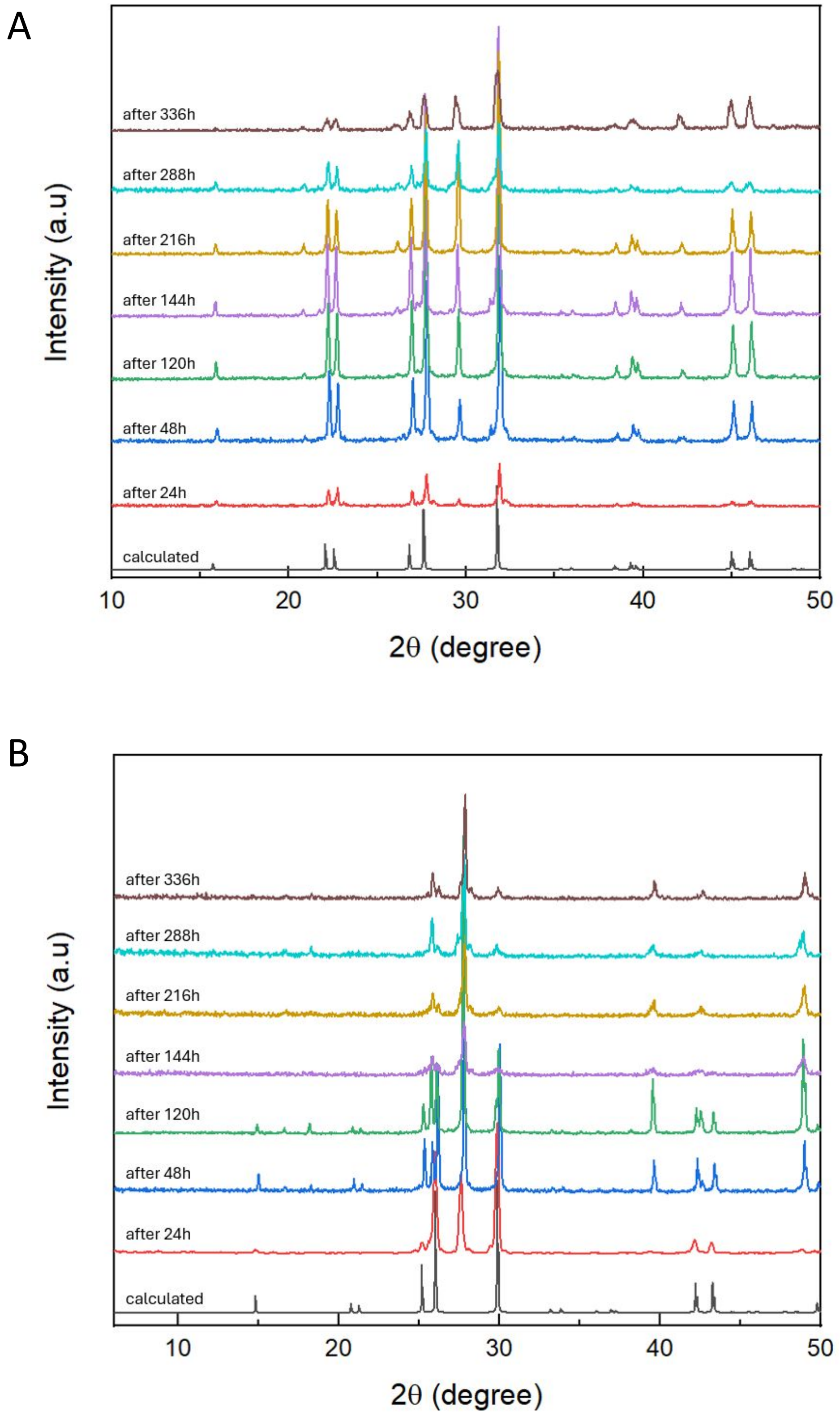

Figure S4. X-ray diffraction patterns of $CsGeX_3$, X= Br (a), I (b) as a function of time. Samples left at open air in the laboratory environment. Bottom pattern in both Figures represent the calculated pattern.

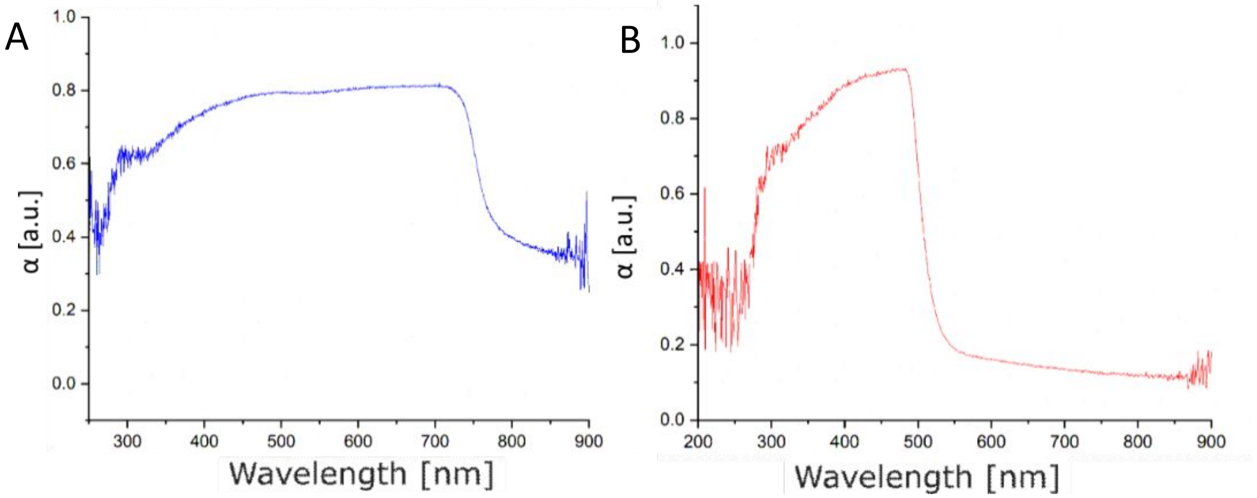

Figure S5. Uv-VIS absorption spectra of powdered samples of (a) $CsGeI_3$ and (b) $CsGeBr_3$.

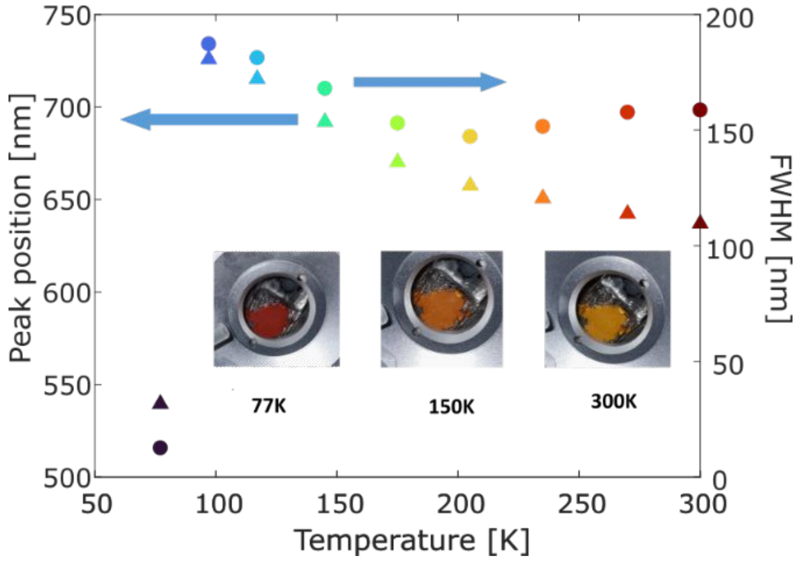

Figure S6. Peak position and FWHM on $CsGeBr_3$ perovskite at different temperature.

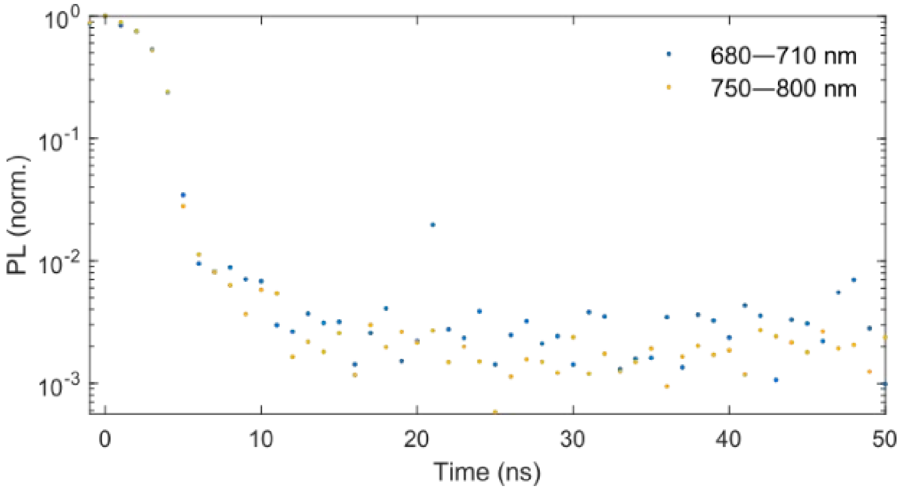

Figure S7. Time resolved photoluminescence measured for the two emission peaks of $CsGeI_3$.

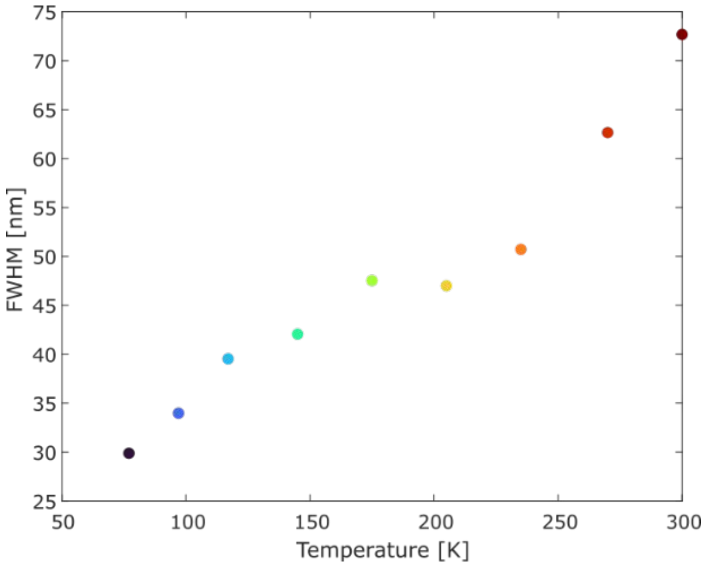

Figure S8. FWHM of $CsGeI_3$ sample at different temperatures.

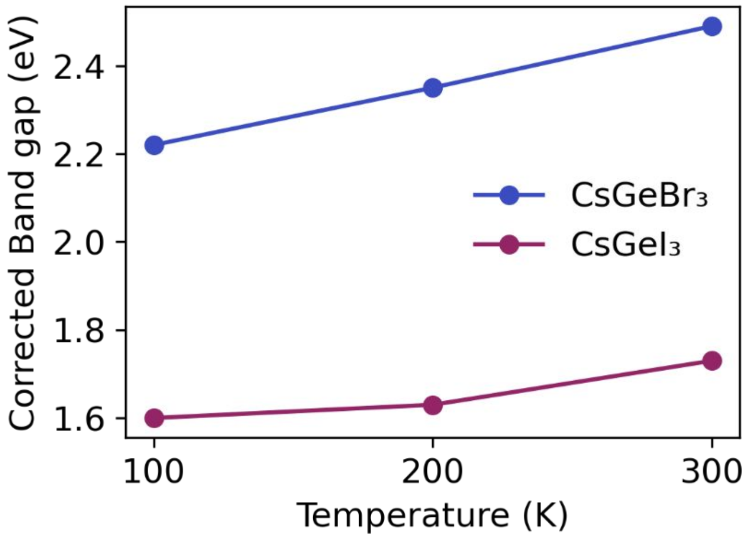

Figure S9. Computed trend of the band gap as a function of temperature for $CsGeBr_3$ and $CsGeI_3$.

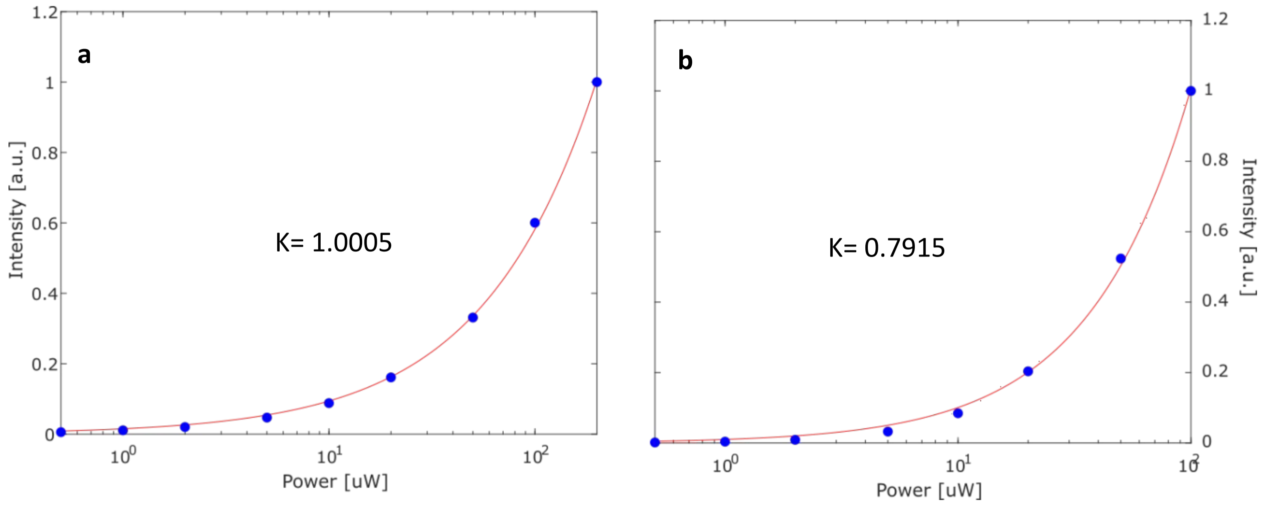

Figure S10. Fluence dependence of the PL intensity on $CsGeBr_3$ sample measured at 97 K fitted with a power law $I=P^k$ for the broad (a) and narrow (b) emission peaks.

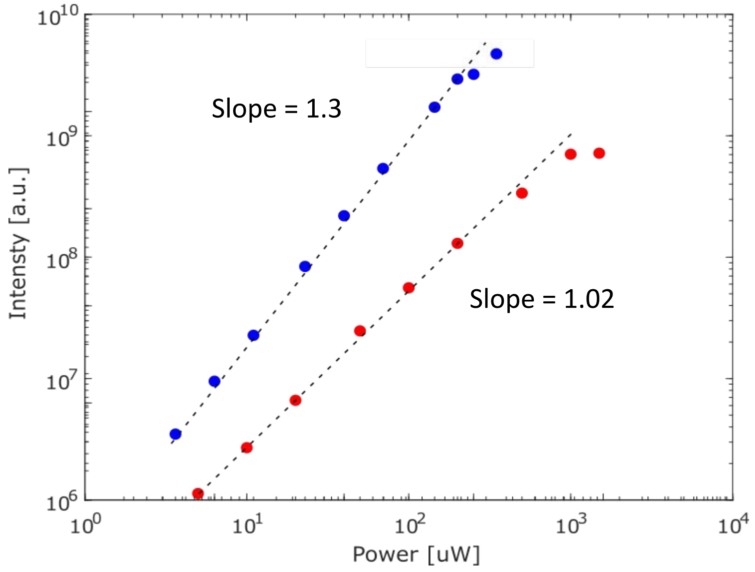

Figure S11. Super linear behavior of the emission intensity of $CsGeI_3$ sample at room temperature (red) and 235 K (blue).

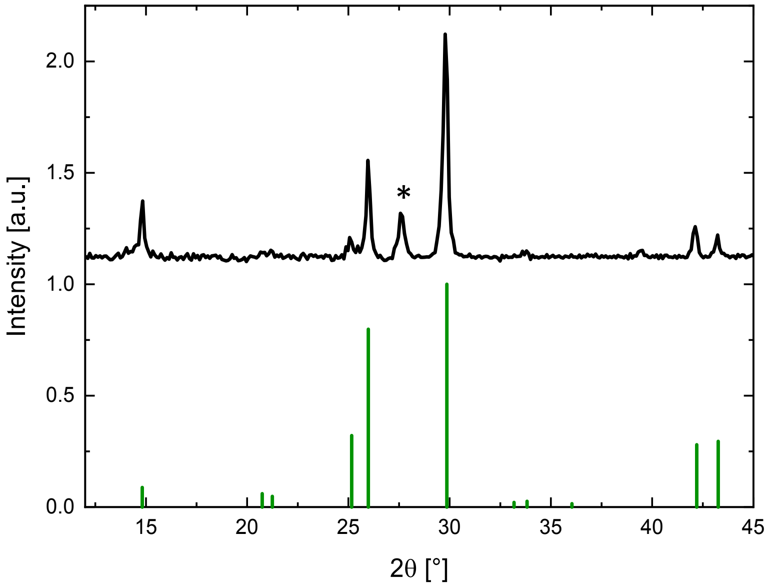

Figure S12. XRD diffractogram of $CsGeI_3$ crystallites is reported in the upper part of the Figure (black line). Vertical green lines represent the calculated pattern of the rhombohedral symmetry. The peak at 27.5° reveal an excess of CsI in the final material (marked with an asterisk).

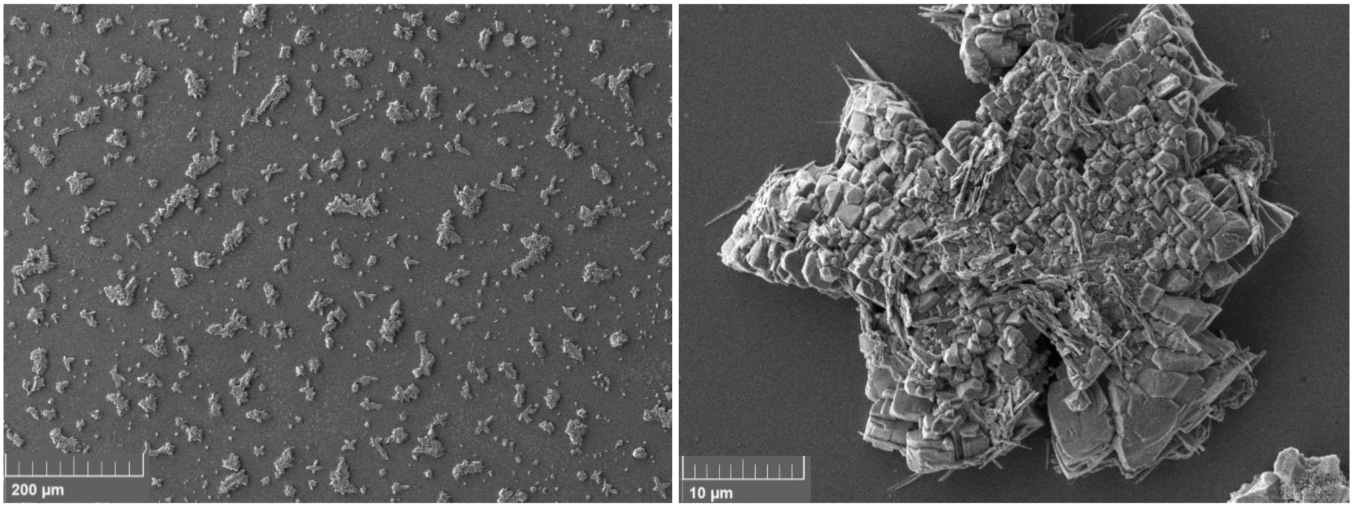

Figure S13. SEM images of $CsGeI_3$ perovskite crystallites deposited on a substrate with a magnification of 283 (left) and 5000 (right).

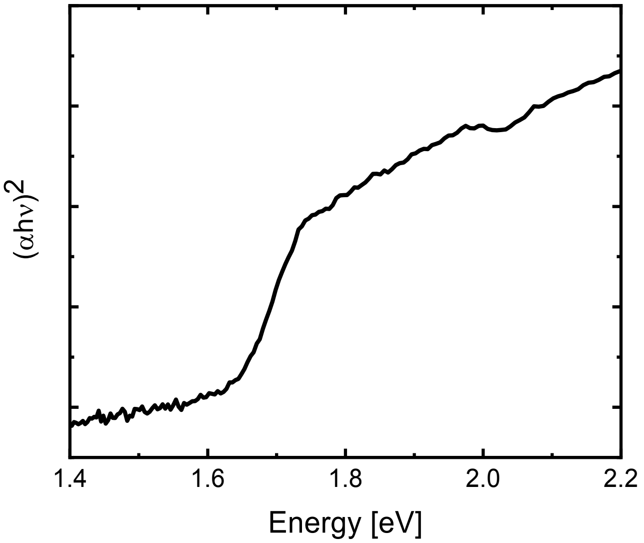

Figure 14 Uv-VIS absorption spectrum of the deposited $CsGeI_3$.

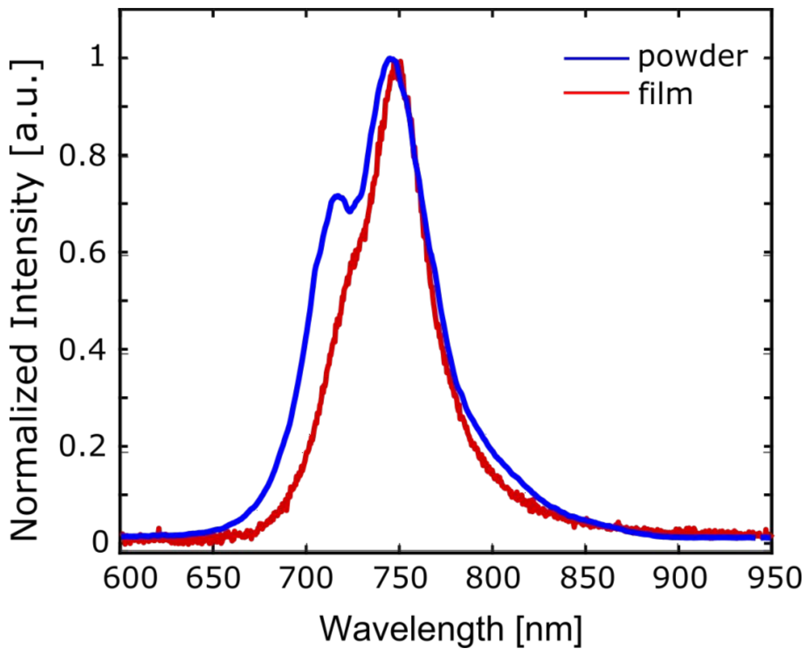

Figure S15. Comparison of the normalized emission spectra of $CsGeI_3$ powder sample (blue) and crystallites (red).
